# The Lipoxygenases: Their Regulation and Implication in Alzheimer’s Disease

**DOI:** 10.1007/s11064-015-1776-x

**Published:** 2015-12-16

**Authors:** Grzegorz A. Czapski, Kinga Czubowicz, Joanna B. Strosznajder, Robert P. Strosznajder

**Affiliations:** Department of Cellular Signalling, Mossakowski Medical Research Centre Polish Academy of Sciences, Pawińskiego 5, 02-106 Warsaw, Poland; Laboratory of Preclinical Research and Environmental Agents, Department of Neurosurgery, Mossakowski Medical Research Centre Polish Academy of Sciences, Pawińskiego 5, 02-106 Warsaw, Poland

**Keywords:** Lipoxygenase, LOX, Alzheimer’s disease, Arachidonic acid, Neurodegeneration

## Abstract

Inflammatory processes and alterations of lipid metabolism play a crucial role in Alzheimer’s disease (AD) and other neurodegenerative disorders. Polyunsaturated fatty acids (PUFA) metabolism impaired by cyclooxygenases (COX-1, COX-2), which are responsible for formation of several eicosanoids, and by lipoxygenases (LOXs) that catalyze the addition of oxygen to linolenic, arachidonic (AA), and docosahexaenoic acids (DHA) and other PUFA leading to formation of bioactive lipids, significantly affects the course of neurodegenerative diseases. Among several isoforms, 5-LOX and 12/15-LOX are especially important in neuroinflammation/neurodegeneration. These two LOXs are regulated by substrate concentration and availability, and by phosphorylation/dephosphorylation through protein kinases PKA, PKC and MAP-kinases, including ERK1/ERK2 and p38. The protein/protein interaction also is involved in the mechanism of 5-LOX regulation through FLAP protein and coactosin-like protein. Moreover, non-heme iron and calcium ions are potent regulators of LOXs. The enzyme activity significantly depends on the cell redox state and is differently regulated by various signaling pathways. 5-LOX and 12/15-LOX convert linolenic acid, AA, and DHA into several bioactive compounds e.g. hydroperoxyeicosatetraenoic acids (5-HPETE, 12S-HPETE, 15S-HPETE), which are reduced to corresponding HETE compounds. These enzymes synthesize several bioactive lipids, e.g. leucotrienes, lipoxins, hepoxilins and docosahexaenoids. 15-LOX is responsible for DHA metabolism into neuroprotectin D1 (NPD1) with significant antiapoptotic properties which is down-regulated in AD. In this review, the regulation and impact of 5-LOX and 12/15-LOX in the pathomechanism of AD is discussed. Moreover, we describe the role of several products of LOXs, which may have significant pro- or anti-inflammatory activity in AD, and the cytoprotective effects of LOX inhibitors.

## Introduction

Aging-related neurodegenerative disorders, including Alzheimer’s disease (AD), have become some of the most important unsolved medical problems. In spite of significant study into the molecular mechanisms of neurodegeneration, there is still no satisfactory efficient treatment, prevention or early diagnosis. Recent studies have suggested that inflammatory processes may play a key role in mechanisms of neurodegeneration. Cyclooxygenases (COX) and lipoxygenases (LOX) are crucial enzymes responsible for the progression of inflammation.

AD, the most common form of dementia, starts many years before the clinical symptoms appear. It was proposed recently that AD progression time may be divided into three phases: damaging phase, where amyloid beta (Aβ) and hyperphosphorylated microtubule-associated protein tau (MAP tau, MAPT) accumulate; synaptic and metabolic alteration phase; and final phase when clinical symptoms may be detected [1, 2]. The neuropathological hallmarks of AD are extracellular deposits of Aβ and intracellular neurofibrillary tangles (NFTs) built of hyperphosphorylated MAPT. Amyloid β and MAPT remain the central focus of AD research, as factors responsible for the activation of the cascade of molecular processes leading to the progression of neurodegeneration.

The amyloid-cascade hypothesis is commonly accepted, but attempts to develop therapeutic methods based on an anti-Aβ approach have not yielded satisfactory results [3–6]. Although experimental and genetic studies have confirmed the key role of amyloidogenesis in AD, the amyloid theory has many weak points, mainly due to the lack of a correlation between the severity of cognitive impairment and the load of senile plaques in the brain. Currently, it is believed that it is not Aβ aggregates in senile plaques but the soluble oligomers that are the most toxic form of Aβ, and this form correlates with dementia [7].

In addition to the accumulation of senile plaques in the brain, a neuropathological hallmark of AD is the neuronal presence of NFT. The degree of cognitive impairment correlates with the severity of the neurofibrillary tangles. The “tau” theory assumes MAPT plays a causative role in AD [8]. The scientific literature provides evidence of both concepts; however, Aβ appears to be responsible for activation of MAPT phosphorylation. The recently proposed “dual pathway hypothesis” assumes that both amyloidogenesis and hyperphosphorylation of MAPT are secondary changes caused by other, upstream factors [9].

A growing body of evidence indicates that inflammatory processes play an important role in the pathomechanism of AD [10–12]. The presence of senile plaques in AD brains induces inflammatory response, leading to activation of microglia and astrocytes and in consequence to increased production of pro-inflammatory mediators and neuronal degeneration and death. It has been demonstrated that activation of complement proteins, cytokines, chemokines, proteases and their inhibitors, proteoglycans, growth factors, and miscellaneous enzymes occurs in the AD [13, 14]. Studies suggest that inflammation contributes significantly to the sporadic form of AD, perhaps even initiating it [15] but inflammation may also exacerbate the progression of AD [16]. However, activation of the phagocytic activity of microglial cells may have also positive effect. Microglia, brain resident macrophages, maintain inflammatory status by secreting cytokines, chemokines and other mediators, as reactive oxygen and nitrogen species that affect surrounding cells. These cells, which constitute 10-15% of all the cells in the brain, may express pro- and anti-inflammatory responses, exhibiting M1 and M2 phenotypes, respectively. The activation of COXs and LOXs in microglia leads to synthesis of huge amounts of metabolites and to release of reactive oxygen species that could affect the function and phenotype of microglia cells. It was recently proposed that anti-inflammatory and pro-resolving lipid mediators such as resolvin D1 and lipoxin A4 may play a role in polarization and maintenance of M2 microglia [17, 18]. Pharmacological inhibition of COX-2 was also shown to affect polarization of peripheral macrophages [19–21]. Microglia express several receptors as RAGE (receptor for advanced glycation endproducts), scavenger receptors SR-AI/II, TLRs (toll-like receptors) TREM2 (triggering receptor expressed on myeloid cells 2) and many others. Most of these receptors are involved in Aβ clearance [22–28]. It is now postulated that if microglia lose their function, the slumbering synapses can be awakened by inflammatory signals evoking massive synapse loss [29]. The last data of Johansson et al. demonstrated that prostaglandin EP2 signaling suppresses beneficial microglia function in AD and that COX/PGE2/EP2 immune pathway could be very promising target(s) to restore microglia function and to prevent AD progression [30].

Epidemiological data indicate that non-steroidal anti-inflammatory drugs (NSAIDs) may have some beneficial effect in AD. In a population-based cohort study of 6989 subjects, an 80% decrease in the risk of developing AD in long-term users of NSAIDs was demonstrated [31]. However, most clinical trials have shown a neutral effect [32] (NCT00004845) or only small beneficial effects of NSAIDs [33] (NCT00065169). The meta-analysis of epidemiological data published in 11 articles indicated that NSAID exposure reduced AD incidence by 58% [34]. It was recently suggested that for a beneficial effect NSAIDs should be administered in the early stages of the disease, in cognitively normal individuals [2]. It is still unclear what the molecular target of NSAIDs is in AD. Possible functional targets are cyclooxygenase-1 and -2 (COX), γ-secretase, Rho-GTPases, and peroxisome proliferator-activated receptors (PPAR) [2].

Also, novel genome-wide association studies (GWAS) have suggested that the innate immune system confers the risk of AD [35, 36]. GWAS studies (done on large cohorts numbering thousands of patients) have indicated that besides the *APOE4* gene, some other genes are also associated with the risk of AD [25, 35, 37–42]. It is supposed that the effect of these genetic factors on the pathomechanism of AD is associated with the regulation of the innate immune system (*ABCA7*, *BIN1*, *CD2AP*, *CD33*, *CLU*, *CR1*, *EPHA1*, *MS4A6A*/*MS4A4E*, *PICALM, TREM2*), and also with the level of Aβ (*ABCA7*, *APOE*, *ATXN1*, *BIN1*, *CD33*, *CLU*, *CR1*, *PICALM, TREM2*), lipid metabolism (*ABCA7*, *APOE*, *CLU*), and signal transduction (*ABCA7*, *BIN1*, *CD2AP*, *CD33*, *CLU*, *EPHA1*, *MS4A6A*/*MS4A4E*, *PICALM, TREM2*) [43]. Although the association of these genes with AD has clearly been confirmed, it is worth noting that their effect on the risk of AD is not high. In most cases, odds ratio (OR) is 1.1–1.2 (compared to *APOE* OR, which is 4 to 15). However, a rare missense mutation (rs75932628-T) in*TREM2* confers increased risk of developing AD with an effect size similar to that for APOE (OR = 2.92) [42]. TREM2 is expressed in microglia and neurons and is involved in promoting phagocytosis and in inhibiting the production of inflammatory mediators by these cells. It is a transmembrane protein that interacts with TYROBP (TYRO protein tyrosine kinase binding protein, Dap12) and forms receptor signaling complex involved in chronic inflammation by triggering the production of constitutive inflammatory cytokines [42]. Recent studies demonstrated that targeting microglial receptors and their signaling pathways may reduce inflammation and Aβ-dependent neurodegeneration.

Neuroinflammation is a double-edged sword that exerts both beneficial and detrimental effects on neurons. The brain’s resident immune cells, microglia, may be protective in AD; however, their improper activation may lead to a worsening of neuronal pathology. Accumulating data highlight the complex nature of these cells [44]. An increasing body of evidence indicates that this phenomenon is related to the variability of phenotypes of immune cells within the brain, but it may also depend on age, stage of the disease and possibly on other factors, including lipid alteration. Most published studies have focused on the role of eicosanoids synthesized by COX-1 and COX-2. The analysis of the effect of these enzymes’ inhibitors is included in the evaluations of NSAIDs actions and their side effects. Published data suggest that lipoxygenases may be involved in pathomechanism of AD. LOXs are key regulators of inflammatory signaling, but may also affect processes directly related to neurotoxic cascades dependent on Aβ and MAPT. In this review, we summarize the role of lipoxygenases, especially 5-LOX and 12/15-LOX, in the pathomechanism of AD. In addition, the neuroprotective effect of LOX inhibitors, as neuroprotectants, is discussed.

## Lipoxygenases

**Fig. 1:**
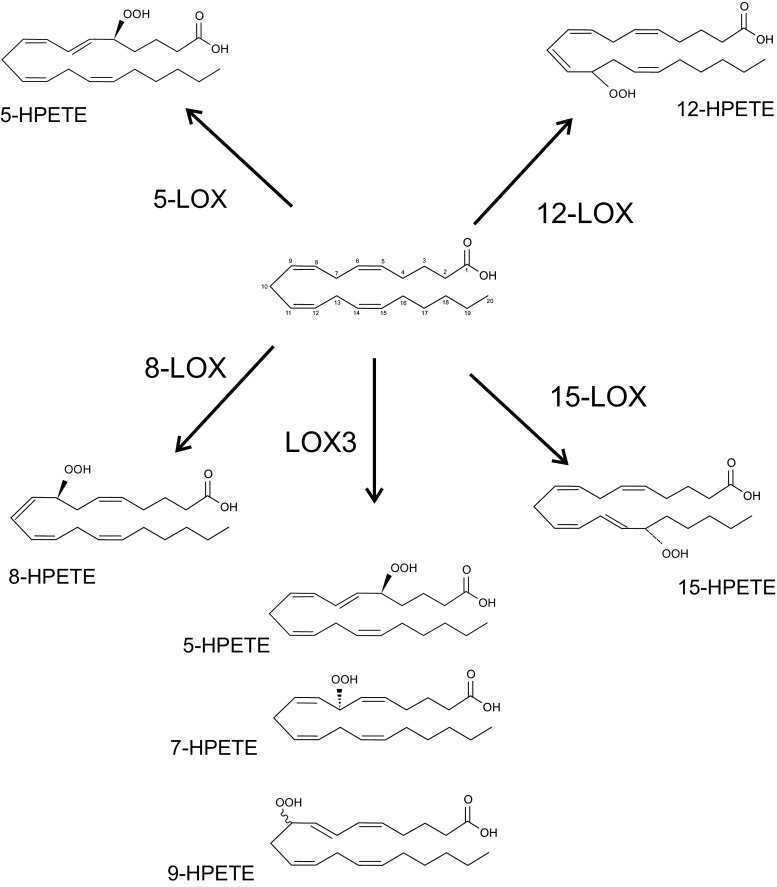
Dioxygenase activity of lipoxygenases. Arachidonic acid was presented as an example substrate

Lipoxygenases (LOXs) are a group of iron-containing dioxygenases that catalyze the stereoselective addition of oxygen to arachidonic acid (AA), docosahexaenoic acid (DHA) and other polyunsaturated fatty acids (PUFA). The basic nomenclature of LOXs (with the exception of LOX-3) is based on the position of the oxygen insertion in a substrate; for example, 5-LOX inserts molecular oxygen into AA to carbon 5 of the aliphatic chain with stereo configuration (Fig. 1). The reaction product of LOX is hydroperoxyeicosatetraenoic acids (HPETE) [45]. LOXs occur in several isoforms according to the type of tissue where they are located, for example reticulocyte type or epidermis type. Some LOXs catalyze several reactions; for example, reticulocyte type LOX inserts molecular oxygen into AA to carbon 12 and 15 in various ratio in different species [46]. There are five types of LOXs in mammalian species: 5-,8-,12-, 15-LOX and LOX-3 [47].

Lipoxygenases are enzymes containing non-heme iron and requiring catalytic activation. This activation process involves transformation of non-active iron in ferrous state Fe^2+^ to iron in ferric form Fe^3+^, accomplished by lipid hydroperoxide oxidation.

**Fig. 2:**
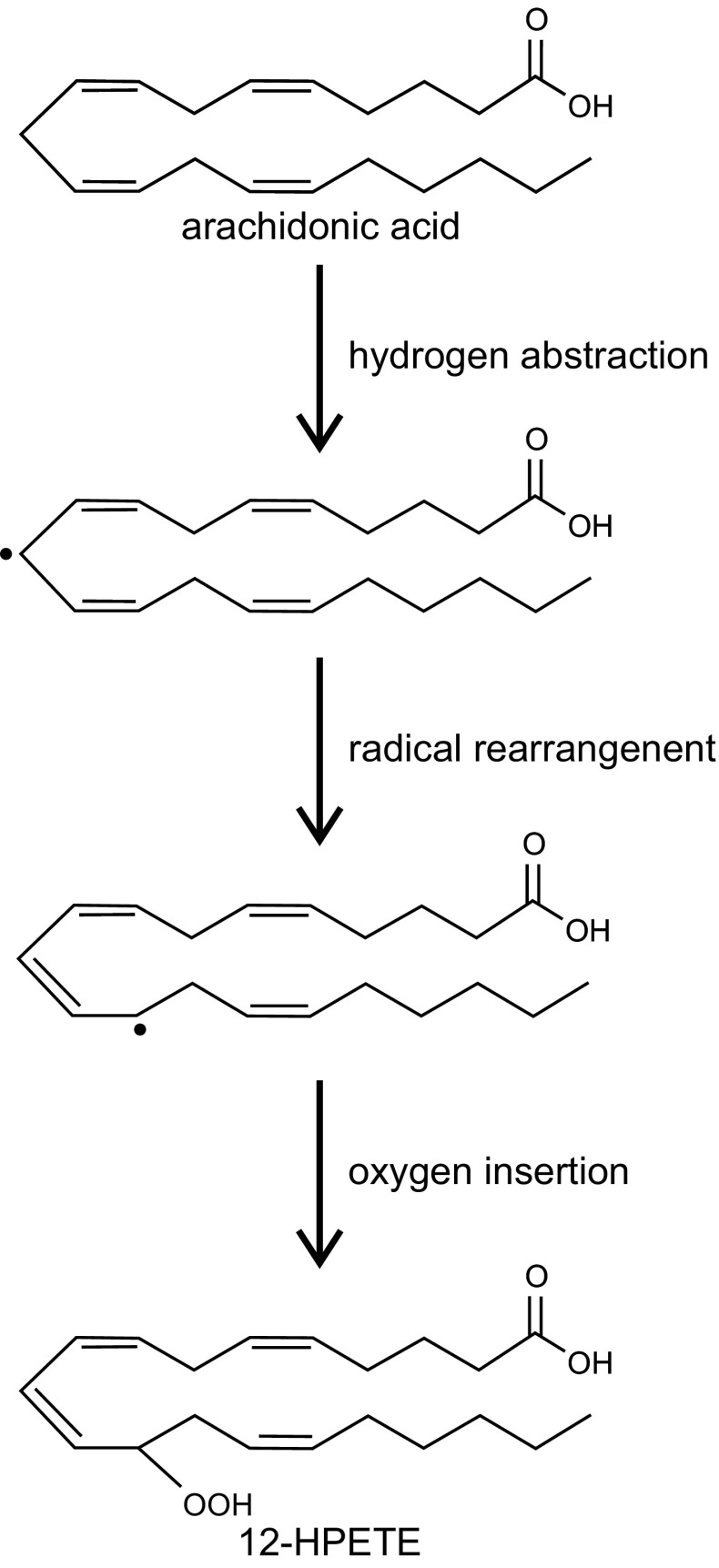
Scheme of LOX reaction (according to [48])

The LOX reaction consists of three consecutive steps (Fig. 2) [48]:

- stereoselective hydrogen abstraction from a bis-allylic methylene group. A carbon–centered fatty acid radical is formed in this process. This process is a rate-limiting step of the LOX reaction,
- radical rearrangement which is accompanied by Z,E–diene conjugation,
- stereoselective insertion of molecular dioxygen and reduction of this hydroperoxy radical intermediate to a corresponding anion and the ferrous LOX is oxidized back to the ferric form.

The products of most mammalian LOXs are usually specific stereoisomers. However, under certain reaction conditions, for example extreme pH or low oxygen concentration, LOXs form complex mixture of sterorandom oxygenation products [48].

The main substrates for LOXs are arachidonic acid and docosahexaenois acid which are released from membrane phospholipids by phospholipases A2 (PLA2) and become accessible to COX and LOX. As AA and DHA share the same enzymes, a competition exists for metabolism, and an excess of one causes substantial fall in the conversion of the other [49]. Cytosolic PLA2 (cPLA2 group IVA-F) was postulated to be more specific for AA-phospholipids and this enzyme could be the predominant in inflammatory signaling [50]. However, Quach et al. presented arguments that secretory sPLA2 is mainly involved in inflammatory processes and the expression of sPLA2 isoforms is changing during progression of several diseases [51]. It seems that some cross-talk exists between cPLA2 and sPLA2, and cPLA2 regulates and enhances activity of sPLA2.

**Fig. 3:**
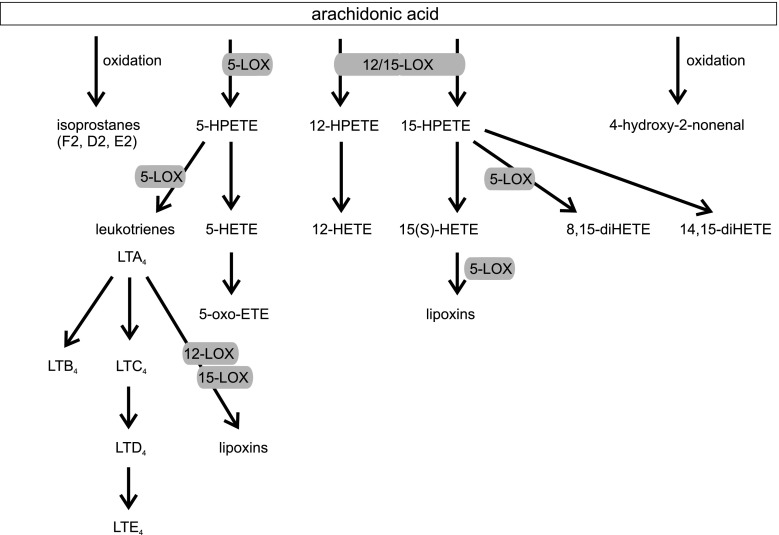
Arachodonic acid cascade—the role of LOX (according to [54])

AA is oxygenated to hydroperoxyl derivatives including HPETEs. These derivatives upon reduction form corresponding hydroxyeicosatetraenoic acids (HETE) and leukotriene (LT) via 5-lipoxygenase, lipoxins and hepoxilins [52–54] (Fig. 3). LOXs peroxidize membrane lipids and lead to structural changes in the cell. The mammalian reticulocyte 15-LOX-1 is the major enzyme which is responsible for membrane lipid peroxidation [55]. This process is significantly increased in aged brains during inflammation and neurodegenerative diseases. Products of LOXs have strong bioactivity properties even in nanomolar concentration; for example HETEs, the metabolites of AA, are ligands of peroxisome proliferator-activated receptors (PPAR) [56]. HETEs also act as secondary messengers in synapses. 5-HPETE inhibits synaptosomal membrane Na^+^, K^+^-ATPase activity [57]. 12-HETE is a neuromodulator the synthesis of which is activated during ischemia. Its role in neurons is the attenuation of calcium influx and glutamate release and the inhibition of AMPA receptor activation [58].

## 5-Lipoxygenase and Its Role in AD

The important lipid peroxidizing enzyme is 5-LOX, which catalyzes the conversion of AA into 5(S)-hydroperoxyeicosatetraenoic acid (5-HPETE) and leukotriene LTA_4_. Further, LTA_4_ is converted into LTB_4_ by LTA_4_ hydrolase or is conjugated with reduced glutathione by LTC_4_ synthase to form LTC_4_ [59]. LTC_4_ is metabolized by elimination of glutamic acid and glycine through the action of a γ-glutamyl-transferase to LTD_4_ and finally LTD_4_ via specific dipeptidase forms LTE_4_ [60]. LTD_4_, LTC_4_ and LTE_4_ are termed cysteinyl LTs (cysLTs) (Fig. 3). Inflammatory eicosanoids which are generated via 5-LOX act on 6 receptors (OXE receptor which recognizes 5-HETE and 5-oxo-ETE, LTB_4_ receptors BLT1 and BLT2, cysteinyl leukotriene receptors CysLT_1_ and CysLT_2_, which recognize leukotrienes LTC_4_, LTD_4_, LTE_4_) [61].

Recent studies have identified a new branch in the 5-LOX pathway of AA metabolism by which 5-oxo-6,8,11,14-eicosatetraenoic acid (5-oxo-ETE) is formed [61]. 5-oxo-ETE is produced from 5-HPETE by the action of 5-hydroxyeicosanoid dehydrogenase (5-HEDH). The actions of 5-oxo-ETE are mediated by the OXE receptor. An extensive review of this area can be found in [62]. Through the combined actions of lipoxygenases the lipoxins are formed [52]. A large number of multicellular responses to injury, inflammation stimuli or infection lead to the formation of lipoxins. Combined activity of 12/15-LOX (human monocytes, macrophages), 5-LOX (neutrophils), or of 5-LOX and platelet 12-LOX (thrombocytes) are involved in lipoxin biosynthesis. The synthesis of lipoxins requires transcellular interaction of various cell types [48].

**Fig. 4:**
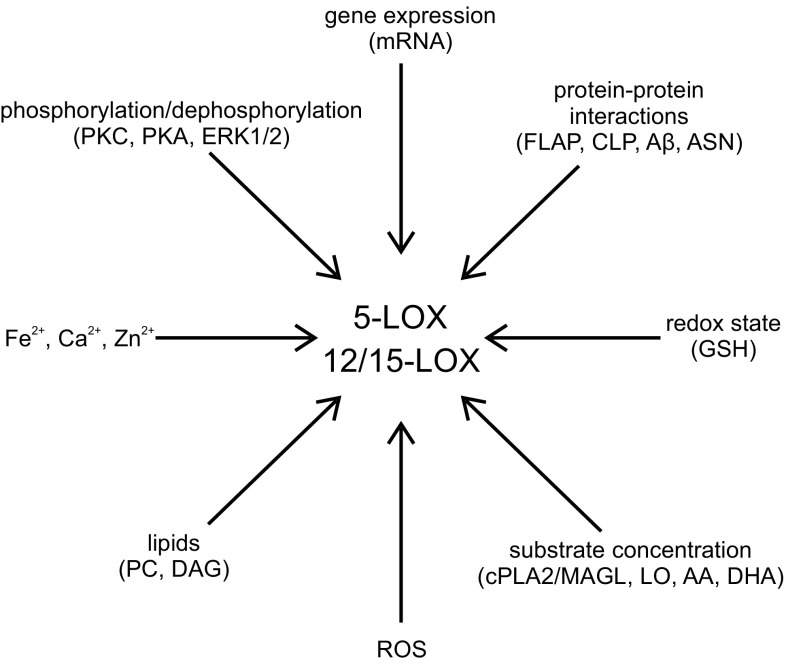
Regulation of 5-LOX and 12/15-LOX

The cellular activity of 5-LOX is regulated in different ways which involve various signaling pathways [59] (Fig. 4). 5-LOX activity can be regulated by an increase in intracellular Ca^2+^ concentration, diacylglycerols, phosphorylation of serine residues (271, 663 and 523) by p38 MAPK and MAPKAP kinases (MK), and extracellular signal-regulated kinase (ERK) 1/2 and protein kinase A (PKA), respectively [59, 63]. The C2-like domain is involved in the Ca^2+^-dependent interaction of 5-LOX with membrane structures. Ca^2+^ stimulates 5-LOX translocation from cytosol to the nucleus. Free AA liberated from phospholipids is transferred by the membrane-bound 5-LOX activating protein FLAP to 5-LOX for further metabolism [59, 64, 65]. Ca^2+^-dependent activation of 5-LOX requires the presence of phosphatidylcholine (PC) or coactosin-like protein (CLP) [66]. Acting together with PC, CLP gives a threefold increase in the amount of LTA_4_ produced by 5-LOX. CLP acts as a scaffold for 5-LOX and also increases the ratio of 5-HETE/5-HPETE [67]. In vitro study has demonstrated that Ca^2+^ is not required for 5-LOX activity in the presence of high concentrations of PC or AA [68]. The stress-induced activation of 5-LOX in human polymorphonuclear leukocytes (PMNL) is Ca^2+^-independent but involves enzyme phosphorylation [69].

5-LOX activity is regulated by phosphorylation. As mentioned above, p38 MAPK, MAPKAP, ERK and PKA are identified as kinases that phosphorylate 5-LOX in vitro at Ser271, Ser663 and Ser523, respectively. The ERKs and p38 MAPKs mediate cellular activation of 5-LOX [70, 71]. ERKs and p38 MAPKs activated by proinflammatory cytokines, chemotactic factors, phorbol esters and Ca^2+^ mobilizing agents, osmotic shock, genotoxic stress, UV light and heat shock induce nuclear translocation of 5-LOX and activation of leukotriene production. The inhibitors of the ERK pathway (SB203580 and U0126) efficiently inhibit AA-induced leukotriene biosynthesis in human polymorphonuclear leukocytes (PMNL) under conditions that do not induce substantial mobilization of Ca^2+^ [59, 69]. An increase in cellular cAMP level leads to phosphorylation of 5-LOX enzyme by PKA, and in consequence to inhibition of 5-LOX activity. The data have shown that PKA activation inhibits 5-LOX translocation and leukotriene biosynthesis in human neutrophils [72, 73].

The redox state is an important parameter of 5-LOX cellular activity. Inhibition of glutathione peroxidases (GPx) or depletion of glutathione leads to an increase in 5-LOX activity [63]. Reduction of lipid hydroperoxides by GPx-1 and GPx-4 inhibits 5-LOX [74]. The catalytic activity of 5-LOX is increased by ATP and, to a lower degree, by other nucleotides, such as ADP, AMP, CTP, UTP and cAMP [63, 75].

5-LOX activity is also regulated by the 5-LOX-activating protein (FLAP). This protein is membrane bound and belongs to the MAPEG (membrane-associated proteins in eicosanoid and glutathione metabolism) protein family. Membrane-embedded FLAP selectively transfers AA to 5-LOX and then AA is oxygenated to 5(S)-HPETE and dehydrated to leukotriene A_4_ (LTA_4_) [76]. FLAP inhibitors may be effectively used to modulate the activity of 5-LOX by the ability to inhibit AA binding to FLAP. Several compounds are able to interact with FLAP, such as MK-0591, Bay-X-1005 and MK-866 [60]. All these inhibitors have good clinical safety profiles and show activity against early and late phases of asthmatic responses and decreases in lung volume after allergen challenge [77].

**Fig. 5:**
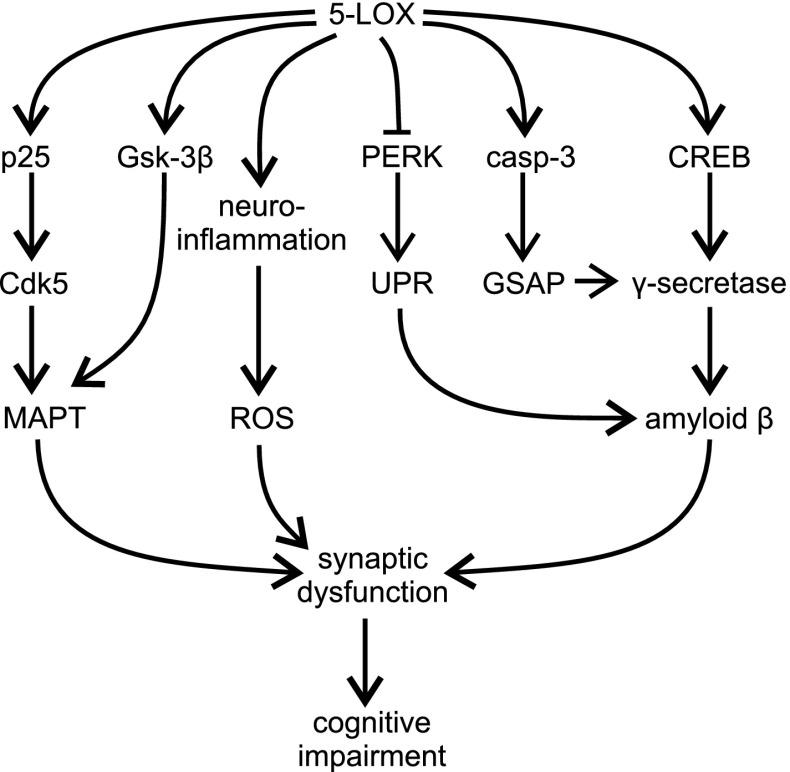
The role of 5-LOX in the pathomechanism of AD

A growing body of evidence indicates that 5-LOX is contributed in the pathomechanism of AD and other aging-associated neurodegenerative disorders (Fig. 5). Aging increases region-specific neuronal expression and activity of 5-LOX in rodents via epigenetic regulation, which may subsequently influence the course of neurodegenerative processes [64, 65, 78–83]. In human brain *post*-*mortem* analysis, it has been demonstrated that intracellular immunoreactivity of 5-LOX is increased in the hippocampus of AD patients, compared to healthy age-matched controls [84, 85]. Double-labeling analysis has demonstrated a close association of 5-LOX immunoreactivity with Aβ plaques, NFTs and vasculature. Interestingly, increased levels of 5-LOX mRNA, protein and activity have recently been demonstrated in peripheral blood mononuclear cells (PBMCs) from late-onset AD (LOAD) patients. Concomitantly, reduced levels of DNA methylation at *ALOX5* promoter have been found [86].

Experiments on animal AD models have provided evidence on the importance of 5-LOX and have demonstrated its role in the pathomechanism of AD. An elevated level of *Alox5* mRNA has been demonstrated in the hippocampus and cortex of Tg2576 mice, a commonly used transgenic (Tg) model of AD. Genetic ablation of 5-LOX in Tg mice (Tg2576) clearly reduced Aβ load in various brain regions, as measured by ELISA, Western blotting and immunohistochemistry [84]. Interestingly, in Tg mice lacking 5-LOX the activity of α- and β-secretase was unaltered, but the activity of γ-secretase was significantly inhibited. The marker of inflammation (TNFα) and oxidative stress (isoprostane F_2α_-III) were also unchanged in 5-LOX deficient mice. Genetic and pharmacological (Zileuton) inhibition of 5-LOX reduced the activity of γ-secretase and the level of Aβ_1–42_ in mouse embryonic fibroblasts (MEFs) [84]. Accordingly, two 5-LOX-derived compounds, 5-HPETE and LTC_4_, increased Aβ_1–40_ production in HEK293 cells stably expressing C99, precursor of Aβ and the immediate substrate for γ-secretase, which suggests a direct effect of 5-LOX on the γ-secretase complex [84]. Moreover, the 5-LOX inhibitor (Zileuton) reduced Aβ formation in these cell lines, supporting the important role of γ-secretase in the amyloidogenic effect of 5-LOX.

A mechanistic explanation of γ-secretase activation by 5-LOX was proposed recently [87–90]. The main product of 5-LOX, 5-HETE, directly activates CREB and promotes its nuclear translocation. CREB has been demonstrated to control expression of all four members of the γ-secretase complex: APH-1, nicastrin, Pen-2 and PS-1. The effect of 5-LOX on amyloidogenesis may be also related to formation of γ-secretase-activating protein (GSAP) [91]. GSAP is a crucial molecule responsible for Aβ production by interacting with the γ-secretase complex. 5-LOX specifically regulates function of GSAP via caspase-3 -catalyzed cleavage of GSAP leading to formation of its active fragment, GSAP 16 kD [90]. Importantly, pharmacological inhibition of 5-LOX in 3xTg mice with Zileuton evokes a specific reduction of γ-secretase complex and consequently decreases the number of Aβ deposits in the brain after 3 months of treatment [92]. In addition, in vitro studies confirmed that Zileuton does not affect Notch signaling [88]. This fact may have an important significance for the potential use of 5-LOX inhibitors in therapeutic applications, because it suggests that by using 5-LOX inhibitors one can avoid the toxic side-effects of inhibition of γ-secretase modulators, which have been observed in the case of the classical inhibitors of γ-secretase. The same effect on γ-secretase function and Aβ load in the brain has been stimulated in a Tg mouse model of AD by indirect inhibition of 5-LOX activity with MK-591, an inhibitor of FLAP [93]. Accordingly, over-expression of 5-LOX in Tg2576 mice evokes a worsening of AD-like phenotype, increasing the level of CREB, PS1, nicastrin, and Pen-2, leading to enhanced accumulation of Aβ in brain tissue and to cognitive impairment [94].

The activity of 5-LOX has also been implicated in MAPT neuropathology. The significant role of 5-LOX has been demonstrated in triple transgenic 3xTg-AD and in Tg2576 mice, which develop amyloid plaques and NFT [95, 96]. Overexpression of 5-LOX by an adeno-associated virus (AAV)-mediated gene transfer evokes γ-secretase-dependent enhancement of Aβ production and plaque formation, as well as cyclin-dependent kinase 5 (Cdk5)-dependent MAPT hyperphosphorylation. Furthermore, activation of neuroinflammatory processes, reduction of synaptic markers, synpatophysin, microtubule associated protein 2 (MAP2) and post-synaptic density protein 95 (PSD-95) and also behavioral deficits have been observed in animals overexpressing 5-LOX. Interestingly, an increased level of p25, a potent activator of Cdk5, was observed. Surprisingly, additional experiments revealed that an inhibitor of γ-secretase reduced Aβ levels, but had no effect on MAPT hyperphosphorylation, indicating that 5-LOX dependent Cdk5-mediated phosphorylation is independent of the Aβ. Moreover, in vitro experiments, using genetic and pharmacological methods of inhibition of Cdk5, have demonstrated that 5-HETE evokes p25-dependent overactivation of Cdk5, which is responsible for the enhancement of MAPT phosphorylation at Ser396/Thr404. It has also been suggested that Gsk-3β is a mediator of 5-LOX-dependent MAPT phosphorylation in Tg2576 mice [97]. Inhibition of 5-LOX activity by the FLAP inhibitor, MK-591, reduces MAPT phosphorylation at Ser396/Ser404, Thr231/Ser235, as well as the level of insoluble MAPT. However, MK-591 has no effect on Cdk5, but reduces Gsk-3β phosphorylation (Ser9) and activity. By using genetic and a pharmacological inhibition of 5-LOX in 3xTg mice, Giannopoulos et al. (2013, 2014) demonstrated an amelioration of synaptic function, integrity and significant improvement of memory [64, 65]. Three month-long treatment with Zileuton reduced Cdk5 activation, MAPT hyperphosphorylation and improved memory dysfunction in aged 3xTg mice [92].

It has also been proposed that 5-LOX may be involved in the regulation of specific pathways of Aβ degradation [98]. The mechanism is related to the PKR-like endoplasmic reticulum kinase (PERK)-dependent pathway of the unfolded protein response (UPR). Accumulation of aggregates of Aβ in the cell increases 5-LOX level and activity, which in turn inhibits the PERK arm of the UPR and enhances the rate of Aβ aggregation. Inhibition of 5-LOX (CNB-001 or BW B70C) or FLAP (MK886) stimulates the PERK/eIF2α/ATF4 pathway leading to Aβ degradation. In a mouse model of AD, inhibition of 5-LOX with CBN-001 reduces Aβ levels, maintains synaptic proteins and reverses cognitive deficits. However, Firuzi et al. [84] demonstrated that the levels of insulin-degrading enzyme and neprilysin, both proteases involved in Aβ catabolism, were not changed in Tg mice lacking 5-LOX.

For a better understanding of the role of 5-LOX in the pathomechanism of AD, the importance of 5-LOX has also been studied in inflammatory reactions, an important component of the pathophysiology of AD. Single intraperitoneal administration of endotoxin lipopolysaccharide (LPS) induces a transient increase in the *Alox5* gene in mouse hippocampus 12–24 h post-injection [99]. By using serial LPS injections to transgenic mice (3xTg) Joshi et al. [83] described that 5-LOX is a crucial player responsible for a worsening of AD-like phenotype by activation of chronic inflammation. LPS treatment had a rather small effect on Aβ level, but significantly exacerbated MAPT pathology in 3xTg mice. This study showed that neuroinflammatory response was reduced in 3xTg mice lacking 5-LOX gene. However, the absence of 5-LOX did not protect against an increase in MAPT phosphorylation at Ser202/Thr205 and Thr231/Ser235. Because the level of p25 increased in the brains of LPS-treated mice, the authors suggested that MAPT phosphorylation evoked by chronic systemic inflammation was catalyzed by Cdk5, and not Gsk-3β [83].

Because psychosocial stress is an important environmental risk factor for AD, in a following study the role of 5-LOX in the corticosteroid-dependent AD-like phenotype was analyzed in vitro and in vivo [100]. Dexamethasone, an anti-inflammatory agent, induced 5-LOX activation and Aβ formation, and pharmacological (AA-861, MK-591) or genetic inhibition of 5-LOX prevented dexamethasone-evoked γ-secretase-dependent increase in Aβ levels. In triple transgenic (3xTg) mice, genetic inactivation of *Alox5* gene, prevented dexamethasone-evoked phosphorylation of specific sites on MAPT and synaptic disruption [101]. It was proposed that Gsk-3β may be responsible for dexamethasone-evoked MAPT phosphorylation.

However, some studies have not confirmed the significance of 5-LOX in the proteotoxicity of Aβ or have given contradictory results. Pharmacological inhibition of 5-LOX (caffeic acid) or downregulation of 5-LOX expression had no effect on Aβ_25–35_-evoked apoptosis in primary rat neurons in vitro [102, 103]. NDGA protected cultured rat hippocampal neurons against the toxicity of Aβ, but gave no protection against Aβ_25–35_-evoked cell death in human neuroblastoma MSN cell cultures [104, 105]. 5-LOX inhibitor (AA861) protected cultured rat hippocampal neurons against Aβ toxicity, whereas the FLAP inhibitor L655,238 was ineffective [104].

## 12/15-LOX and Its Role in AD

The most abundant LOX isoforms in the central nervous system (CNS) are 12/15-LOX. The metabolites of these enzymes, 12(S)-HETE and 15(S)-HETE, play an important role as secondary messengers in synaptic transmission and are involved in learning and memory processes. 12/15-LOX have been described abundantly in neurons and in some glial cells throughout the cerebrum, hippocampus and basal ganglia [106, 107]. Oxidative stress mechanisms and inflammatory reactions have been involved in the up-regulation of 12/15-LOX activity and expression levels [106, 108] (Fig. 4).

15-LOX-1 (12/15-LOX) preferentially metabolizes linoleic acid to 13-hydroperoxydecadienoic acid (13-HODE), but also arachidonic acid to 15-HETE and to 12-HETE [109] (Fig. 3). Hepoxilins are the products of AA metabolized through the 12S-LOX pathway. Hepoxilins are bioactive epoxy-hydroxy eicosanoids. After oxygenation of AA by 12S-LOX 12S-HPETE is formed. Then, the pathway is divided into two branches, on the one hand 12S-HPETE is reduced to 12S-HETE or on the other hand 12S-HPETE is converted to bioactive hepoxilin A_3_ [53]. Various types of cells can form hepoxilins, for example platelets [110], neutrophils [111], and brain cells [112]. Hepoxilins, especially HXA_3_, are involved in many biological processes, for example in the regulation of membrane permeability, calcium transport, insulin secretion, and chemotaxis [111, 113]. Zafiriou et al. [113] showed that HXA_3_ upregulates mRNA and protein expression of phospholipid peroxide glutathione peroxidase (PhGPx). The study by Pallast et al. [114] suggested that 12/15-LOX mediated neuronal cell death by glutathione depletion evoked by extracellular glutamate in HT22 cells. Wang et al. [115] showed that 12-LOX activation plays a key role in oxidative injury due to glutathione (GSH) depletion caused by cysteine deprivation in premyelinating oligodendrocytes (preOLs) and mature OLs. Inhibiting this enzyme with AA-861 effectively protected OLs from GSH depletion and also blocked the accumulation of ROS induced by cysteine deprivation. It has also been suggested that zinc can participate in the activation of 12-LOX leading to free radical formation and neuronal injury [116]. Under pathological conditions, zinc enters postsynaptic neurons through NMDA receptors, calcium permeable AMPA/kainate receptors and also voltage sensitive calcium channels [117]. Oxidative stress causes zinc release, which activates 12-LOX [116, 118]. *N*,*N*,*N*′,*N*′-tetrakis(2-pyridylmethyl) ethylenediamine (TPEN) a zinc chelator effectively blocks the activity of 12-LOX [116].

The first direct evidence indicating that the 12-LOX metabolic pathway is altered in AD was demonstrated in *post*-*mortem* analysis by Pratico et al. [119], who showed an increase in 12/15-LOX protein level, in the frontal and temporal cortex. Consequently, the level of 12-HETE and 15-HETE, two products derived from the activation of 12/15-LOX, was elevated in these brain structures. The increase correlated with the level of a specific marker of lipid peroxidation, the isoprostane 8,12-iso-iPF2_α_VI, and inversely correlated with the level of vitamin E, suggesting the prominent role of 12/15-LOX as a generator of oxidative stress. The increase in the level of 12(S)-HETE and 15(S)-HETE, which correlated with lipid peroxidation (isoprostane 8,12-iso-iPF2αVI) and MAPT protein levels, has also been observed in the cerebrospinal fluid (CSF) of AD patients [108]. Moreover, the levels of 12(S)-HETE and 15(S)-HETE are also elevated in CSF of individuals with mild cognitive impairment (MCI), suggesting that the 12/15-LOX pathway plays an important role during the initiation and early phase of AD. In accordance, absence of 12/15-LOX reduces oxidative stress in the CNS of ApoE-deficient mice [106].

**Fig. 6:**
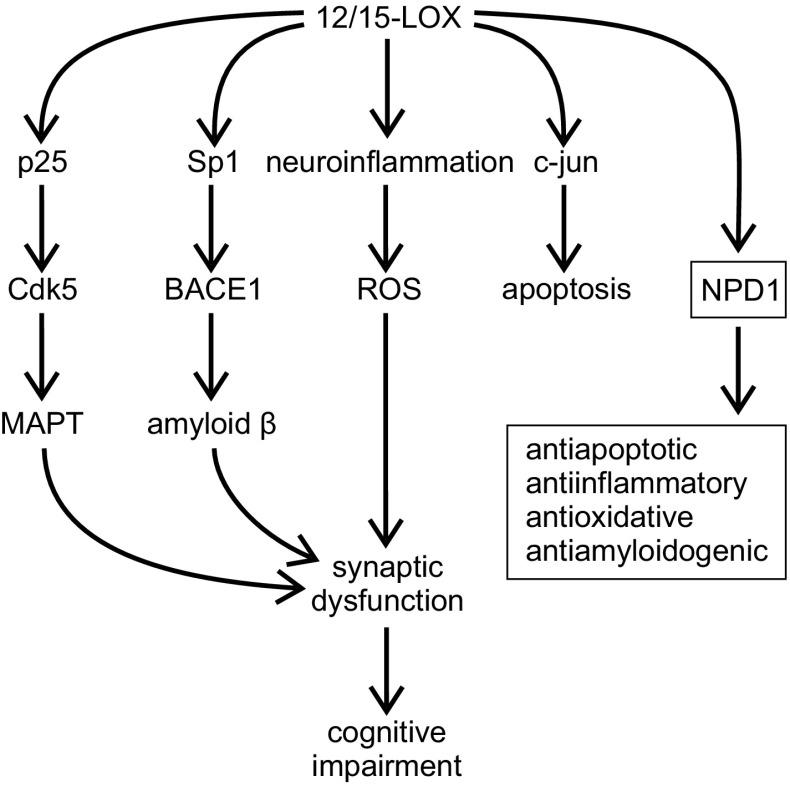
The role of 12/15-LOX in the pathomechanism of AD

12/15-LOX participates in the processing of Aβ by modulating the BACE1 proteolytic cascade [120, 121] (Fig. 6). Accordingly, deletion of 12/15-LOX gene reduced the generation of Aβ, and overexpression of 12/15-LOX increased its level. The active contribution of 12/15-LOX in Aβ formation was correlated with the level of the BACE1 protein. The significance of 12/15-LOX has been confirmed in vivo. In a transgenic mouse model of AD (Tg2576), a significant upregulation of 12/15-LOX expression and activity occurs, followed by an increase in lipid and protein oxidation, as compared to WT mice [122]. Genetic deletion of 12/15-LOX gene reduces BACE1 levels, as well as, Aβ_1–40_ and Aβ_1–42_ load in the hippocampus and cortex of Tg2576 mice, indicating the crucial role of 12/15-LOX in amyloidogenesis. Similar effects were achieved by treatment with a specific 12/15-LOX inhibitor, PD146176, which efficiently attenuated BACE1 pathway leading to significant reduction in Aβ levels and substantial improvement of memory function in 3xTg mice [123]. 12/15-LOX activates BACE1 by increasing mRNA and protein level via transcription factor Sp1 [121]. Importantly, in mice devoid of 12/15-LOX, the reduction of cognitive deficits has been observed. Consequently, overexpression of 12/15-LOX in Tg2576 mice evokes activation of astrocytes and microglia cells, alterations of brain synaptic integrity and worsening results in contextual and cued fear conditioning tests, suggesting both hippocampal and amygdala impairment [121, 124]. In neuronal cells stably expressing human Swedish mutant APP (N2A-APPswe), specific metabolites produced by 12/15-LOX (12(S)/15(S)-HETE) induce an increase in BACE1 expression and activity, without any impact on APP or ADAM10. Two selective and structurally different inhibitors of 12/15-LOX (PD146176 and CDC) reduce the level of Aβ secreted by cells stably expressing human APP. Baicalein improves cognition in Aβ-evoked toxicity in mouse and increases cell survival in APPsw-expressing PC12 cells subjected to oxidative stress [125, 126]. However, due to potent antioxidative properties, non-specific effects of baicalein must be taken into consideration [127, 128].

It has been observed that genetic or pharmacological (baicalein) inhibition of 12-LOX prevents c-jun dependent apoptosis evoked by Aβ_25–35_ in primary cultures of rat cortical neurons [102, 103]. Accordingly, 12(S)-HETE induces an increase in c-jun expression and apoptosis.

12/15-LOX also contributes to MAPT pathology. The level of insoluble fraction of MAPT and the level of phosphorylation of MAPT at Ser396/Thr404 (PHF-1), Ser396 (PHF13), Ser202/Thr205 (AT8), Thr231/Ser235 (AT180) was significantly reduced in 3xTg mice treated with 12/15-LOX inhibitor, PD146176 [123]. Overexpression of 12/15-LOX increases phosphorylation of MAPT at Ser202/Thr205 and Ser396 both in the brains of Tg2576 mice and in N2A (neuro-2 A neuroblastoma) cells stably expressing human APP bearing double Swedish mutation [124]. A significant increase in the level and activity of Cdk5, but not Gsk-3β was observed. Moreover, pharmacological (roscovitine) or genetic inactivation of Cdk5 efficiently prevented MAPT phosphorylation, indicating that this phosphorylation was specifically mediated by activity of Cdk5.

**Fig. 7:**
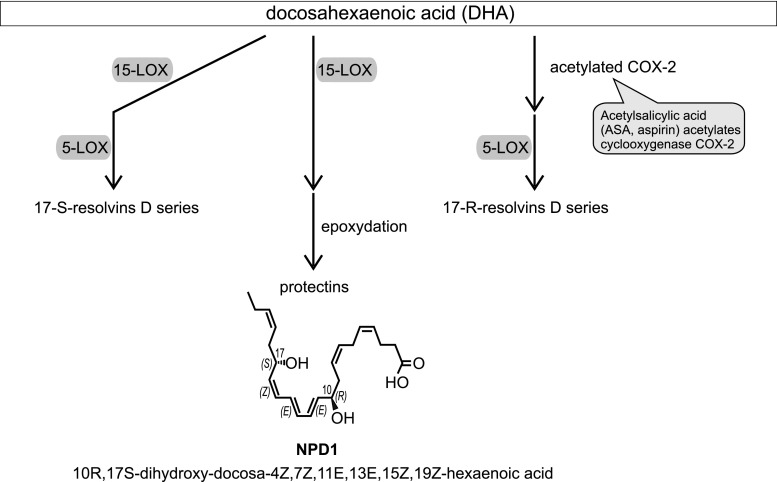
DHA cascade—the role of LOX (according to [130, 146, 147])

However, the important role of LOX in the pathomechanism of AD has also been related to the brain cell survival pathway via involvement in the synthesis of antiapoptotic and neuroprotective docosahexaenoic acid (DHA)-derived 10R,17S-dihydroxy-docosa-4Z,7Z,11E,13E,15Z,19Z-hexaenoic acid, known as neuroprotectin D1 (NPD1) (Fig. 7) [129–131]. Due to an increase in the expression of NF-κB-sensitive miRNA-125b, the level of 15-LOX mRNA is reduced in hippocampi of AD patients [132]. Consequently, the level of NPD1 was decreased. As NPD1-induced gene expression regulates secretion of Aβ, and has antiapoptotic and anti-inflammatory effects, inhibition of cPLA2/15-LOX-dependent pro-survival pathway could lead to a worsening of AD-related pathology [133]. Synthetic, exogenous NPD1 down-regulates inflammatory signaling, amyloidogenic APP cleavage and apoptosis in cells overexpressing APPsw or exposed to oligomeric Aβ peptide [134].

## Summary

Among the many pro-oxidative pathways, COX and LOX seem to be especially important in AD-related pathology, including inflammatory processes. Consumption and metabolism of AA by the brain is up-regulated in AD patients, suggesting that AA is involved in the pathomechanism of this disease [135]. Accordingly, the level of many enzymes responsible for AA metabolism (e.g. cPLA2, sPLA2, iPLA2, COX-1, COX-2, mPGES-1, 12-LOX, 15-LOX, cPGES-1, p450 epoxygenase) is changed in postmortem AD brain samples [136]. The alterations of the above enzymes lead to oxidative stress and in consequence to free radical-dependent DNA damage and poly(ADP-ribose) polymerase-1 (PARP-1) overactivation, neuronal degeneration and death. Also, our study demonstrated that inflammatory processes significantly affect PARPs and NADPH oxidase expression [137]. The main enzymes liberating AA and DHA in basal and inflammation conditions in the nervous system are cPLA_2_ and monoacylglycerol lipase (MAGL) [138, 139]. It has been proposed that enzymes located downstream, e.g. LOX and COX, and specific receptors for selected eicosanoids should offer promising targets for therapy.

Epidemiological studies have suggested that anti-inflammatory therapy may be effectively used to prevent, treat, or slowdown the progression of AD [140, 141]. However, chronic treatment with COX inhibitors appears ineffective, at least in middle to late stages of AD. Moreover, serious gastrointestinal and cardiovascular side-effects of anti-COX therapy reduce the usefulness of this therapeutic strategy. Recently, it was suggested that 5-LOX-mediated metabolism of AA may contribute to the side-effects evoked by NSAIDs [142]. The conventional NSAIDs are inhibitors of COX-2 and COX-1, but have no effect on enzymatic activity of 5-LOX, therefore they disturb the balance between COXs and LOXs. This situation leads to enhancement of 5-LOX activity and to accumulation of leukotrienes LTC_4_, LTD_4_ and LTE_4_. These compounds, in addition to their well-known potent bronchoconstrictor properties, together with prostanoids are potent mediators of the main phenomena of inflammation, such as vascular changes, increase in body temperature and leucocyte migration. Other data have demonstrated that downregulation of 5-LOX improves synaptic function and memory in animal models of AD. Compounds inhibiting both COX and LOX, so-called “dual inhibitors” or “multiple target inhibitors,” could show improved side-effect profiles [142, 143]. For these all reasons dual COX and LOX inhibition is probably better than inhibition of one pathway.

Moreover, it was recently suggested that, for beneficial effect, NSAIDs should be administered in early stages of AD (phase 1), in cognitively normal individuals. Unfortunately, it is still unclear what the molecular target of NSAIDs in AD is. Possible targets are COX-1 and COX-2, γ-secretase, Rho-GTPases, NADPH oxidase and PPAR [2]. It seems that a better understanding of the role of LOX in the pathogenesis of AD might enable the development of far more effective disease-modifying approaches based on inhibitors of LOX. Finally, in our attempts to use LOX inhibitors and other anti-inflammatory compounds in AD we should consider the cytoprotective potential of inflammation. Additionally, the heterogeneity of individual inflammatory response must also be analyzed [144, 145].
